# Lethal, Sub-Lethal and Trans-Generational Effects of Chlorantraniliprole on Biological Parameters, Demographic Traits, and Fitness Costs of *Spodoptera frugiperda* (Lepidoptera: Noctuidae)

**DOI:** 10.3390/insects13100881

**Published:** 2022-09-28

**Authors:** Zunnu Raen Akhtar, Ayesha Afzal, Atif Idrees, Khuram Zia, Ziyad Abdul Qadir, Shahbaz Ali, Inzamam Ul Haq, Hamed A. Ghramh, Yasir Niaz, Muhammad Bilal Tahir, Muhammad Arshad, Jun Li

**Affiliations:** 1 Department of Entomology, University of Agriculture Faisalabad, Faisalabad 38000, Pakistan; 2 Institute of Molecular Biology and Biotechnology, The University of Lahore, 1-Km Defense Road, Lahore 54000, Pakistan; 3 Guangdong Key Laboratory of Animal Conservation and Resource Utilization, Guangdong Public Laboratory of Wild Animal Conservation and Utilization, Institute of Zoology, Guangdong Academy of Sciences, Guangzhou 510260, China; 4 Office of Research, Innovation & Commercialization (ORIC), University of Agriculture Faisalabad, Faisalabad 38000, Pakistan; 5 Honeybee Research Institute, National Agricultural Research Centre, Park Road, Islamabad 45500, Pakistan; 6 Department of Entomology and Wildlife Ecology, University of Delaware, Newark, DE 19716, USA; 7 Department of Agricultural Engineering, Khwaja Fareed University of Engineering and Information Technology, Rahim Yar Khan 64200, Pakistan; 8 Research Center for Advanced Materials Science (RCAMS), King Khalid University, P.O. Box 9004, Abha 61413, Saudi Arabia; 9 Department of Physics, Khwaja Fareed University of Engineering and Information Technology, Rahim Yar Khan 64200, Pakistan

**Keywords:** *Spodoptera frugiperda*, chlorantraniliprole, demographic parameters, fitness costs, two-sex life table

## Abstract

**Simple Summary:**

This is the first study providing important time-specific, age-specific, and reproduction-specific data for managing *Spodoptera frugiperda* infestations in maize crops using chlorantraniliprole. The application of chlorantraniliprole insecticide suppressed the population of *S. frugiperda*. The results revealed that fecundity was affected by chlorantraniliprole in the second filial generation, which suggests that the insecticide application during spring will prevent *S. frugiperda* infestation in maize crops during the autumn season.

**Abstract:**

Fall armyworm [*Spodoptera frugiperda* (J. E. Smith, 1797)] was first reported in the Americas, then spread to all the continents of the world. Chemical insecticides are frequently employed in managing fall armyworms. These insecticides have various modes of actions and target sites to kill the insects. Chlorantraniliprole is a selective insecticide with a novel mode of action and is used against Lepidopteran, Coleopteran, Isopteran, and Dipteran pests. This study determined chlorantraniliprole’s lethal, sub-lethal, and trans-generational effects on two consecutive generations (F_0_, F_1_, and F_2_) of the fall armyworm. Bioassays revealed that chlorantraniliprole exhibited higher toxicity against fall armyworms with a LC_50_ of 2.781 mg/L after 48 h of exposure. Significant differences were noted in the biological parameters of fall armyworms in all generations. Sub-lethal concentrations of chlorantraniliprole showed prolonged larval and adult durations. The parameters related to the fitness cost in F_0_ and F_1_ generations showed non-significant differences. In contrast, the F_2_ generation showed lower fecundity at lethal (71 eggs/female) and sub-lethal (94 eggs/female) doses of chlorantraniliprole compared to the control (127.5–129.3 eggs/female). Age-stage specific survival rate (*S_xj_*), life expectancy (*E_xj_*) and reproductive rate (*V_xj_*) significantly differed among insecticide-treated groups in all generations compared to the control. A comparison of treated and untreated insects over generations indicated substantial differences in demographic parameters such as net reproduction rate (R_0_), intrinsic rate of increase (r), and mean generation time (T). Several biological and demographic parameters were shown to be negatively impacted by chlorantraniliprole. We conclude that chlorantraniliprole may be utilized to manage fall armyworms with lesser risks.

## 1. Introduction

The fall armyworm [*Spodoptera frugiperda* (*S. frugiperda* hereafter)] is a devastating pest in most of the tropical and subtropical Americas [1,2]. In Africa, it has become a noxious agricultural pest [3,4]. Commonly affected crops by *S. frugiperda* include corn, rice, sugarcane, sorghum etc. [5,6]. It may have several broods yearly and covers great distances in a single night’s flight. The larvae of the pest eat the leaves, stalks, and flowers of cultivated plants [1,7]. The *S. frugiperda* populations are often controlled using chemical pesticides [8,9]. Different types of insecticides work in different ways to kill the target organisms [10]. Most pesticides are neurotoxic due to their effects on acetylcholine receptors (e.g., neonicotinoids), acetylcholine esterases (e.g., carbamates), or ion channel activity in nerve cell membranes (pyrethroids) [11]. Few insecticides act on chitin biosynthesis (benzoylurea, buprofezin), juvenile hormone (phenoxyphenoxy ether), or ecdysone to affect insect growth and molting (triazine). Other insecticides damage the midgut membrane or act on the mitochondrial respiratory electron transport chain (e.g., carbamates) (toxin of *Bacillus thuringiensis*) [11]. Different insecticides, such as emmamectin benzoate [12] and neem extracts [13], have been used to control *S. frugiperda*. 

The baseline susceptibilities of deltamethrin, chlorantraniliprole, flubendiamide, thiodicarb, and chlorpyrifos have been determined against *S. frugiperda* [14]. Similarly, the baseline susceptibilities of different insecticides with control failure estimation for *S. frugiperda* were determined in Burkina Faso [15]. Entomopathogenic nematodes were used to control *S. frugiperda* in Thailand [16]. Insecticide ‘ampligo’ was used against *S. frugiperda* in the coastal Savannah agroecological zone of Ghana [17]. Different insecticides having field efficacy against *S. frugiperda* have been tested [18,19,20]. However, the lethal, sub-lethal and trans-generational effects of chlorantraniliprole on biological parameters, demographic traits, and fitness costs of *S. frugiperda* have been less explored in Pakistan.

Anthranilic diamides have a unique mode of action that activates the unregulated release of internal calcium storage channels, resulting in the depletion of calcium from an insect body, ultimately leading to paralysis and insect death [21]. Chlorantraniliprole belongs to anthranilic insecticides and is registered against Lepidopteran, Coleopteran, Dipteran, and Hemipteran insects [22,23]. Insecticides exert sub-lethal impacts on insects depending upon exposure time and dose [5,24,25]. Due to these sub-lethal impacts, insects experience minor effects on fecundity, reproduction, and development [26]. In addition to the lethal effects (direct killing), insecticides can also result in the degradation and chemical distribution in the field, negatively impacting insect physiology, behavior, reproduction, longevity and biology [23,27,28,29]. Chlorantraniliprole showed toxicity and field efficacy against *S. frugiperda* [30]. It also showed effective control against *S. frugiperda* when used through drip irrigation in China [31]. Chlorantraniliprole also provided effective control over the pest when used in combination with other pesticides/plant extracts [32]. Similarly, chlorantraniliprole showed toxicity in combination with carbaryl against *S. frugiperda* [33]. In the same way, chlorantraniliprole showed toxicity against *S. frugiperda* when combined with neem extract [34]. Different insecticides, including chlorantraniliprole showed sublethal effects on the development and reproduction of *S. frugiperda* [12].

Insect mortality, fertility, and lifespan may all be affected by environmental variables, including heat, pesticides, and secondary plant metabolites. Demographic toxicology, or the life table, is useful for assessing these impacts [35,36,37,38]. The conventional life table focused only on the female population and overlooked the male population. Furthermore, it does not consider data about individual variations and developmental phases [39]. Age-stage two-sex life tables eliminated the inherent inaccuracies present in life tables based on females by adding data from both sexes of a community into their calculations [40,41].

Understanding these population dynamics, which may assist explain distinct sub-lethal consequences on target insects, can be aided using the age-stage two-sex life table [42,43]. Knowing the population dynamics of certain insect species is important for the timely implementation of integrated pest control, two-sex tables with sub-lethal doses may serve this purpose [44,45]. 

Numerous studies implemented the two-sex life table for this purpose. For example, the development and reproduction of *S. frugiperda* were studied by Xie et al. [46] using an age-stage, two-sex life table to see how the effects of various hosts (maize and kidney bean) affected the organism. Guo et al. determined the larval performance and oviposition of *S. frugiperda* using two sex tables on three host plants [47]. The fitness and population life tables of *S. frugiperda* on solanaceous and oilseed crops have been determined in earlier studies [48,49]. Using a two-sex life table, sub-lethal effects of spinetroam against *S. frugiperda* growth and fecundity were determined [50]. Similarly, Iqbal et al. [51] used an age-stage, two-sex life table to investigate the impact that zinc oxide generated in the culture supernatant of *B. thuringiensis* had on the demographic characteristics of *Musca domestica*. Likewise, an age-stage, two-sex life table analysis was used to assess the predatory functional response and fitness characteristics of *Orius strigicollis* Poppius-fed *Bemisia tabaci* and *Trialeurodes* *vaporariorum* [52]. In the same way, ecotoxicological experiments were used to examine the sub-lethal effects of propargite on *Amblyseius swirskii* (Acari: Phytoseiidae) utilizing an age-stage, two-sex life table [53]. Various control measures for the management of arthropod pests are now being developed by researchers. These tactics are aimed to be less harmful to humans, the environment, and predators [54,55,56,57,58]. However, synthetic insecticides are still among the best options available.

The current study aimed to identify the lethal, sublethal, and transgenerational effects of chlorantraniliprole on *S. frugiperda* in Pakistan. Determining the lethal concentration and its impact on all larval instars of *S. frugiperda* survival will be helpful in understanding its chemical control in a better way. The impacts of sub-lethal concentrations on development, reproduction, and fecundity till two generations will help to overcome future resistance development in the maize cropping systems. A two-sex life table will help understand the control of *S. frugiperda* during its all larval, pupal, and adult exposure involving both the male and female sexes, which will further help control it under field conditions.

## 2. Materials and Methods

### 2.1. Field Insect Collection

For laboratory studies, the insects were collected from the research fields of the University of Agriculture in Faisalabad, Pakistan (31°26′15.2″ N 73°04′37.9″ E) and were kept in cages. Insecticide-free maize leaves were given for colony preparation, and the adults were fed with a 10% honey solution. The studied species is an agricultural pest; therefore, no ethical permissions were required for the study.

### 2.2. Bioassay for Larvae

A bioassay study was conducted on newly hatched larvae using the leaf dip method. Maize leaves were cut into 6 cm discs and dipped in insecticide for 20 s. Chlorantraniliprole was added to distilled water according to the chosen concentrations. A preliminary test to find the dilution was conducted, and concentration was chosen accordingly. Leaves were dried after soaking and placed individually in Petri dishes. Each treatment was repeated three times, and mortality was observed after 48 h.

### 2.3. Lethal and Sub-Lethal Effects of Chlorantraniliprole on F_0_, F_1_ and F_2_ Generations

Lethal and sub-lethal concentrations were used in this experiment to observe mortality, survival, development duration (larva, pupa, and adult), fecundity, and reproductive parameters of *S. frugiperda*. Leaves were dipped in lethal concentration solutions (Table 1) of insecticide for 20 s. An untreated control was also included in the study for comparison. One larva was released in each Petri dish, and observations were taken after 48 h. Mortality was recorded, and surviving larvae were fed with fresh leaves of maize. For pairing the insects, pupae were taken to other dishes, differentiated during the pupal stage, and released pairwise in Petri dishes. Cotton soaked in a honey solution was placed inside the vial. The pairs were observed daily for their fecundity.

### 2.4. Transgenerational Effects of Chlorantraniliprole on F_1_ and F_2_ Generations

Ninety (90) eggs were placed in an insect breeding chamber at 27 ± 1 °C and 75% relative humidity for each treatment to observe the transgenerational effects of chlorantraniliprole on the F_1_ and F_2_ generations of *S. frugiperda*. Upon hatching, one larva was placed in each Petri dish for observation and fed with insecticide-dipped leaves. The leaves were dipped in insecticide for 20 s, dried and provided to the larvae for feeding. Later, fresh leaves were changed every 24 h. The developmental period and survival rate of males and females were recorded.

### 2.5. Statistical Analysis

Concentrations (LC_10_, LC_25_, LC_50_, and LC_90_) that caused 10%, 25%, 50%, and 90% mortality were calculated using POLO-Plus [59]. The data on mortality were examined using a one-way analysis of variance, and the mean differences were determined using Tukey’s HSD test in SAS software [60] at 95% probability. Using a two-sex table [42], and TWO-SEX MS CHART Program [61], we were able to assess many biological and fitness characteristics, as well as survival rate, adult lifespan, and age-specific fertility. Bootstrap analysis with a sample size of 10,000 was used to assess the means and standard errors of various life and biological parameters [62]. A confidence interval of difference was used to calculate the results of the bootstrap and paired bootstrap tests [63]. Age-stage specific survival rate (*s_xj_*), age-stage specific net reproductive value (*v_xj_*), and age-stage specific survival rate (*e_xj_*) were determines according to Chi [42]. To create the graphs for the demographic factors, SigmaPlot version 12.0 was used.

The following equations were used to construct the age-stage component of the two-sex life table l_x_:

$$l_{x}=\overset{k}{\underset{j=1}{\sum }}s_{xj}$$

where k is the last stage of the study cohort.

Similarly, age-specific fecundity (m_x_) was calculated as follows:

$$m_{x}=\frac{\sum_{j=1}^{k}s_{\mathrm{xj}}f_{\mathrm{xj}}}{\sum_{j=1}^{k}s_{\mathrm{xj}}}$$

According to Goodman’s recommendation, the Euler–Lotka equation was used to determine the intrinsic rate of rise [64].

$$\overset{\infty }{\underset{x=0}{\sum }}e^{-r\left(x+1\right)}l_{x}m_{x}=1$$

The R_0_ (net reproductive rate), which is the total number of offspring that an individual can produce during the lifetime, was calculated as:

$$R_{0}=\overset{\infty }{\underset{x=0}{\sum }}l_{x}m_{x}$$

The relationship between R_0_ and mean female fecundity (F) was calculated as:

$$R_{0}=F\;\frac{\mathrm{Nf}}{N}$$

The N in the above equation represents the total number of individuals, while f presents the number of female adults in the study [65].

The finite rate (λ) was recorded as:

λ = e^r^

The mean generation time (T) presents the time span that the population needs to increase R_0_ folds of its size. The value of T was calculated as follows:

$$T=\frac{{\mathrm{lnR}}_{0}}{r}$$

Age-stage life expectancy (e_xj_) was calculated as follows:

$$e_{\mathrm{xj}}=\overset{\infty }{\underset{i=x}{\sum }}\overset{\beta }{\underset{y=j}{\sum }}s_{\mathrm{iy}}^{\prime }$$

where s_iy_ is considered as probability, an individual of x and j will survive to age i and stage and calculated by the equation below:

$${S^{\prime }}_{\mathrm{iy}}=1$$

Age-stage reproductive value is (V_xj_) defined as the contribution of individuals of age x and stage j for the future population of insects. For age stage-specific, two-sex tables, the following equation is used [66] and calculated as follows:

$$V_{\mathrm{xj}}=\frac{e^{r\left(x+1\right)}}{s_{\mathrm{xj}}}\overset{\infty }{\underset{i=x}{\sum }}e^{-r\left(x+1\right)}\overset{\beta }{\underset{y=j}{\sum }}s_{\mathrm{iy}}^{\prime }f_{\mathrm{iy}}$$

## 3. Results

### 3.1. Toxicity of Chlorantraniliprole to F_0_, F_1_, and F_2_ Generations

The lowest (1.432 mg/L) and the highest LC_50_ (4.119 mg/L) value in F_0_ generation was recorded for the first and sixth instar larvae, respectively. Similarly, the lowest (0.810 mg/L) and the highest (4.080 mg/L) LC_50_ values of the F_1_ generation were noted for the first and sixth instar larvae, respectively. A similar trend for the LC_50_ value was noted for the F_2_ generation. The lowest (0.829 mg/L) and the highest (4.10 mg/L) LC_50_ value of the F_2_ generation was observed for the first and sixth instar larvae, respectively (Table 1).

The LC_10_ and LC_25_ values were determined from mortality concentration-response lines. The lowest (1.042 mg/L) and the highest (1.413 mg/L) LC_10_ value of the F_0_ generation was noted for the first and sixth instar larvae, respectively. Similarly, the first and sixth instar larvae of the F_1_ generation recorded the lowest (0.334 mg/L) and the highest (1.055 mg/L) LC_10_ values, respectively. Moreover, a similar trend in the LC_10_ value was observed of the F_2_ generation, where the first and sixth instar larvae had the lowest (0.345 mg/L) and the highest (1.315 mg/L) LC_10_ values, respectively (Table 1).

The lowest (1.212 mg/L) and the highest (2.345 mg/L) LC_25_ values of the F_0_ generation were noted in the first and sixth larval instars, respectively. Similarly, the first and sixth instar larvae of the F_1_ generation recorded the lowest (0.516 mg/L) and the highest (2.002 mg/L) LC_25_ values, respectively. A similar trend of LC_25_ values was noted for the F_2_ generation, where the lowest (0.52 mg/L) and the highest (2.25 mg/L) LC_25_ values were recorded for the first and sixth larval instars, respectively (Table 1).

The lowest (1.969 mg/L) and the highest (12.012 mg/L) LC_90_ values of the F_0_ generation were recorded for the first and sixth larval instars, respectively. Similarly, the first and sixth instar larvae of the F_1_ generation recorded the lowest (1.908 mg/L) and the highest (15.776 mg/L) LC_90_ values, respectively. A similar trend of LC_90_ values was noted for the F_2_ generation, where the lowest (1.99 mg/L) and the highest (12.82 mg/L) LC_90_ values were recorded for the first and sixth larval instars, respectively (Table 1).

### 3.2. Sub-Lethal and Transgenerational Effects of Chlorantraniliprole on Biological and Reproductive Parameters and of F_0_, F_1_ and F_2_ Generations

The LC_10_ and LC_25_ concentrations of chlorantraniliprole were used to observe biological and reproductive parameters on all instars and pupae in F_0_, F_1_, and F_2_ generations (Table 2 and Table 3).

Significant differences were noted among LC_10_ and LC_25_ concentrations and control treatment of the study. There was no significant difference in hatching duration of F_0_ (*F =* 6.40; df = 89; *p =* 0.214), F_1_ (*F =* 5.45; df = 89; *p =* 0.205) and F_2_ (*F =* 0.63; df = 89; *p* = 0.65) generations (Table 2). Significant difference was recorded for the first instar larval duration of F_0_ (*F =* 8.92; df = 89; *p =* 0.000124) generation, but not for the F_1_ (*F =* 2.79; df = 89; *p =* 0.066) generation. Similarly, F_2_ generation (*F =* 0.28; df = 89; *p* = 0.123) remained non-significant in this regard (Table 2). Significant differences were noted in 2nd instar larval duration of F_0_ (*F =* 4.43; df = 89; *p =* 0.0152) and F_1_ (*F =* 4.21; df = 89; *p =* 0.010) generations, whereas non-significant differences were noted for the F_2_ generation (*F =* 0.25; df = 89; *p* = 0.089) (Table 2). For 3rd instar larvae, non-significant differences were observed in larval duration of F_0_ (*F =* 9.75; df = 89; *p =* 0.088); however, in F_1_ (*F =* 9.56; df = 89; *p =* 0.024) generation significant difference was observed, while non-significant differences were noted for the F_2_ generation (*F =* 10.70; df = 89; *p* = 0.94) (Table 2).

The fourth instar larvae noted significant differences for larval duration in F_0_ (*F =* 2.78; df = 89; *p =* 0.0037), but non-significant for F_1_ (*F =* 3.83; df = 89; *p =* 0.384) and significant differences for the F_2_ generation (*F =* 2.48; df = 89; *p* = 0.007) (Table 2). Significant differences were observed in the larval duration of fifth instars belonging to the F_0_ (*F =* 1.85; df = 89; *p =* 0.013), F_1_ (*F =* 1.89; df = 89; *p =* 0.011) generations; however, non-significant differences were recorded for the F_2_ (*F =* 3.14; df = 89; *p* = 0.068) generation (Table 2). Similarly, significant differences were observed in the larval duration of sixth instars belonging to F_0_ (*F =* 4.42; df = 89; *p =* 0.0009), whereas those belonging to F_1_ (*F =* 1.23; df = 89; *p =* 0.26) and F_2_ (*F =* 1.93; df = 89; *p* = 0.20) generations remained non-significant (Table 2). For pupa duration, non-significant differences were noted in F_0_ (*F =* 1.39; df = 89; *p =* 0.243), F_1_ (*F =* 0.51; df = 89; *p =* 0.646) and F_2_ (*F =* 1.30; df = 89; *p* = 0.36) generations (Table 2).

Reproductive parameters of the F_0_, F_1_ and F_2_ generations are given in Table 3. Pre-oviposition for F_0_ had non-significant differences (*F =* 0.955; *p* = 0.523), while significant differences were noted for F_1_ (*F =* 3.27; *p* = 0.0032) and F_2_ (*F =* 3.61; *p* = 0.00040) generations. Fecundity for F_0_ (*F* = 1.738; *p* = 0.991), F_1_ (*F* = 1.390; *p* = 0.172) and F_2_ (*F* = 0.295; *p* = 0.284) observed non-significant differences. Female adult longevity for F_0_ (*F* = 0.412; *p* = 0.368), F_1_ (*F* = 0.095; *p* = 0.468) and F_2_ (*F* = 1.894; *p* = 0.943) remained non-significant (Table 3).

### 3.3. Effect of Chlorantraniliprole on Demographic Traits of F_0_, F_1_, and F_2_ Generations

Demographic characters calculated using two sex stage-specific life tables are shown in Table 4. For the F_0_ generation, the intrinsic rate of increase (*r*) was directly proportional to concentration which significantly decreased in LC_10_ and LC_25_ compared to the control (Table 4).

The finite mean rate of increase (*λ*) was significantly different in LC_10_ and LC_25_ compared to the control (Table 4) and changed with increased concentration. The net reproductive rate (*R_0_*) was higher in control and decreased significantly with increased concentration in LC_10_ and LC_25_. The mean generation time (*T*) was prolonged in LC_10,_ and LC_25_ treated insects compared to the control (Table 4). The *GRR* was significantly low in LC_10_ and LC_25_-treated insects compared to the control (Table 4).

For the F_1_ generation, *r* was directly proportional to concentration which significantly decreased in LC_10_ and LC_25_ compared to the control (Table 4). The *λ* was significantly different in LC_10_ and LC_25_ compared to the control (Table 4) and changed with increased concentration. The *R_0_* was higher in control and decreased significantly with increased concentration in LC_10_ and LC_25_. The *T* was prolonged in the LC_10_ and LC_25_-treated insects compared to the control (Table 4). The *GRR* was significantly low in the LC_10_ and LC_25_-treated insects compared to the control (Table 4).

For the F_2_ generation, *r* was directly proportional to concentration which significantly decreased in LC_10_ and LC_25_ compared to the control (Table 4). The *λ* was significantly different in LC_10_ and LC_25_ compared to the control (Table 4) and changed with increased concentration. The *R_0_* was higher in control and decreased significantly with increased concentration. The *T* was prolonged in LC_10_ and LC_25_-treated insects compared to the control (Table 4). The *GRR* was significantly low in LC_10_ and LC_25_-treated insects compared to the control (Table 4).

**Figure 1:**
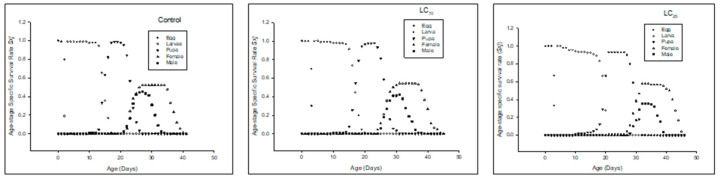
Age stage-specific survival rate (*s_xj_*) of the F_0_ generation in *Spodoptera frugiperda*.

Age-stage specific survival rate (*s_xj_*) of the F_0_ generation denoted that the overall life span of the F_0_ (filial generation) prolonged in LC_10_ and LC_25_ as compared to the control (Figure 1).

**Figure 2:**
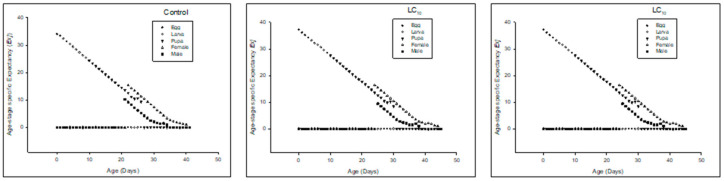
Age stage life expectancy (*e_xj_*) of the F_0_ generation in *Spodoptera frugiperda*.

Age-stage-specific life expectancy(*e_xj_*) was higher in LC_10_ and LC_25_-treated insects than in the control (Figure 2).

**Figure 3:**
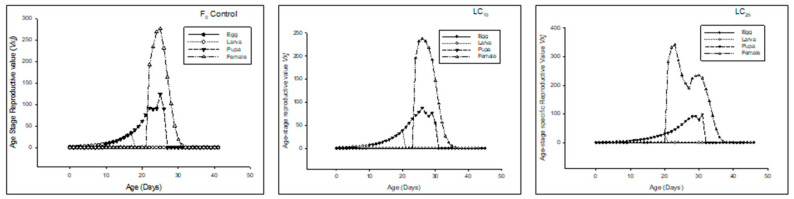
Age stage reproductive value (*v_xj_*) of the F_0_ generation in *Spodoptera frugiperda*.

Age-stage specific reproductive rate (*v_xj_*) of the F_0_ generation denoted that the overall reproductive rate reduced in LC_25_-treated insects, and the LC_10_-treated insects also had less reproductive rate as compared to the control (Figure 3).

**Figure 4:**
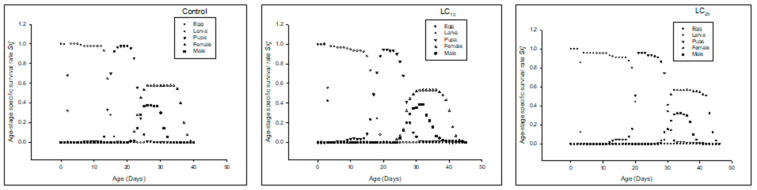
Age stage-specific survival rate (*s_xj_*) of the F_1_ generation in *Spodoptera frugiperda*.

The *s_xj_* of F_1_ (first filial generation) denoted that the overall life span was prolonged in LC_10_ and LC_25_ compared to the control (Figure 4).

**Figure 5:**
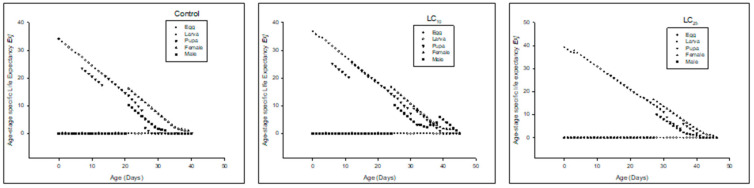
Age stage life expectancy (*e_xj_*) of the F_1_ generation in *Spodoptera frugiperda*.

The *e_xj_* was higher in LC_10,_ and LC_25_-treated insects compared to the control (Figure 5).

**Figure 6:**
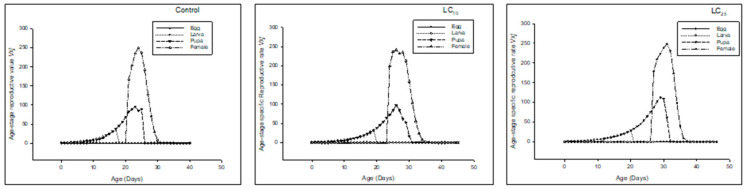
Age stage reproductive value (*v_xj_*) of the F_1_ generation in *Spodoptera frugiperda*.

The *v_xj_* of the F_1_ generation denoted that the overall reproductive rate was reduced in LC25-treated insects, and LC_10_-treated insects had less reproductive rate as compared to the control (Figure 6).

**Figure 7:**
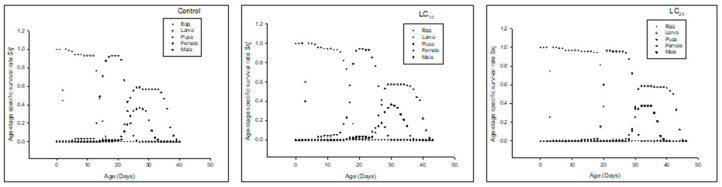
Age stage-specific survival rate (*s_xj_*) of the F_2_ generation in *Spodoptera frugiperda*.

The *s_xj_* of F_2_ (second filial generation) denoted that the overall life span was prolonged in LC_10_ and LC_25_ compared to the control (Figure 7).

**Figure 8:**
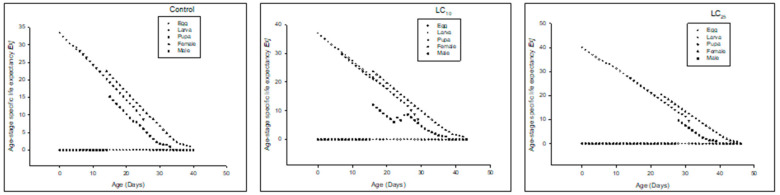
Age stage life expectancy (*e_xj_*) of the F_2_ generation in *Spodoptera frugiperda*.

The *e_xj_* was higher in LC_10_ and LC_25_-treated insects than in the control (Figure 8).

**Figure 9:**
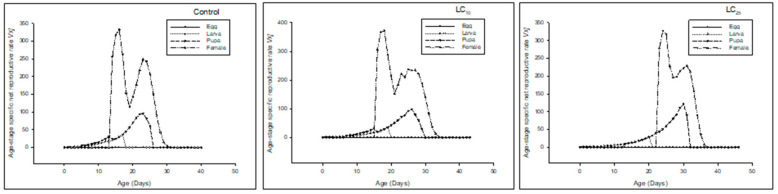
Age stage-specific reproductive rate (*v_xj_*) of the F_2_ generation in *Spodoptera frugiperda*.

The *v_xj_* of the F_2_ generation denoted that the overall reproductive rate was reduced in LC_25_-treated insects, and LC_10_-treated insects had less reproductive rate as compared to the control (Figure 9).

## 4. Discussion

By comprehending the life table of insects, effective management techniques may be created to control insects that are infesting agricultural plants. A greater understanding of the life cycle, survival rate, and reproduction may aid in managing insect pests [67,68]. In the context of muscle function, chlorantraniliprole is an anthranilic diamide that acts as a target for ryanodine receptors. After ingesting anthranilic pesticides, insects experience calcium loss, which leads to muscular contractions. 

According to the current research findings, exposure to sublethal quantities of chlorantraniliprole led to a considerable reduction in both fecundity and fertility (egg hatch). On the other hand, Teixeira et al. [69] found that eating chlorantraniliprole at a concentration of 500 mg L^−1^ did not have a significant impact on the quantity of eggs deposited by apple maggot fly or the percentage of eggs that hatched. Knight and Flexner [70] similarly found that chlorantraniliprole had only a little impact on the adult *C. pomonella* population’s capacity to survive and reproduce. It is possible that the varying quantities of pesticides used cause variations between earlier and current findings, the various species of insects tested, and the technique used to apply the pesticides. Aside from that, the sublethal concentrations of chlorantraniliprole significantly extended the preoviposition of adults. This was in agreement with the observations made by Teixeira et al. [69], which stated that chlorantraniliprole-exposed insects begin egg-laying later than non-exposed adults do.

In accordance with the findings of Han et al. [71], who found that fecundity was dramatically decreased in LC_10_ and LC_30_-treated groups in comparison to the control group, our findings show that fecundity was severely reduced. Similar results were seen in our experiments, in which groups treated with LC_10_ and LC_25_ had a considerably lower fecundity than the control. Our findings are in further accord with Lutz et al. [72], who found that the lifespan of larvae and pupae was far longer than previously estimated. In the same way, the duration of the larval and pupal stages was lengthened in LC_10_- and LC_25_-treated groups compared to the control group in the present research. It’s possible that the disruption to the ryanodine receptors caused the patient to stop eating, which contributed to the protracted duration. Our findings are similarly in accordance with those of Ali et al. [5], who found that the development stages of the larval and pupal stages were severely altered in comparison to the control. 

Compared to the control group, the length of time spent as a larva in the group of insects that had been treated with chlorantraniliprole for the present research was much longer. However, in our studies, pupal and adult emergence were not significantly altered in chlorantraniliprole-sprayed insects as compared to the control. Similar results have been reported for *S. exigua* where chlorantraniliprole decreased larval weight, pupal weight, and pupation rate. Nawaz et al. [73] reported that R_0_, *r*, and λ significantly decreased in chlorantraniliprole-treated groups compared to the control. Similar results for these parameters were recorded in the current study.

Similarly, Han et al. [71] observed a reduced survival rate and less fecundity in chlorantraniliprole-treated insects compared to control. Our study also recorded a lower survival rate and less fecundity in the chlorantraniliprole-treated insects compared to the control. Similar findings have also been reported by Wang et al. [74], where early-instar larvae of *P. xylostella* were affected more at 14 DAT when exposed to chlorantraniliprole-treated radish seedlings using the field rate. Long-lasting residual efficacy of chlorantraniliprole has also been observed against other pests like oblique banded leafroller [75], the grapevine moth and white grubs.

According to Han et al. [71], the values of R, r, and λ were considerably lower in chlorantraniliprole-treated groups compared to the control. These metrics showed a considerable drop in severity in the groups treated with chlorantraniliprole, which produced similar results as seen in the present investigation. According to Fernandes et al. [76], sublethal poisoning might affect an insect’s overall fitness and its reproductive capabilities. This notion was reinforced by the findings of the current study with *P. xylostella*. Yin et al. [77] reported quite similar findings to these, and observed that sublethal doses of Spinosad inhibited the population growth of *P. xylostella* by impairing the organism’s ability to survive, develop, and reproduce.

## 5. Conclusions

This is the first study that provides important basic time-specific, age-specific, and reproduction-specific data for understanding a *S. frugiperda* attack on maize with chlorantraniliprole. The impacts on their development and fecundity resulted in a decreased population of *S. frugiperda*. The results revealed that fecundity was mainly affected by chlorantraniliprole in the second filial generation, which suggests that chlorantraniliprole spraying in the spring season will save maize crops from *S. frugiperda* during the autumn, which is as the main attacking season of the fall armyworm.

## Figures and Tables

**Table 1:** Toxicity of chlorantraniliprole on six larval instars of the F_0_, F_1_ and F_2_ generations of *Spodoptera frugiperda*.

Generation	LC_10_ (mg/L)	LC_25_ (mg/L)	LC_50_ (mg/L)	LC_90_ (mg/L)	Slope ± SE	X^2^	*p*-Value	df
First instar								
F_0_	1.04 (0.86–1.16)	1.21 (1.06–1.31)	1.43 (1.32–1.51)	1.96 (1.84–2.17)	9.27 ± 1.05	33.16	2.07	16
F_1_	0.33 (0.26–0.42)	0.51 (0.41–0.61)	0.81 (0.69–0.92)	1.90 (1.68–2.19)	3.44 ± 0.16	53.50	3.34	16
F_2_	0.34 (0.26–0.41)	0.52 (0.43–0.60)	0.82 (0.72–0.93)	1.99 (1.76–2.30)	3.36 ± 0.14	58.60	3.66	16
Second instar								
F_0_	1.10 (0.89–1.22)	1.25 (1.08–1.34)	1.44 (1.33–1.51)	1.88 (1.76–2.15)	11.02 ± 1.57	36.36	2.27	16
F_1_	0.37 (0.27.46)	0.54 (0.43–0.65)	0.84 (0.71–0.96)	1.92 (1.69–2.22)	3.58 ± 0.17	59.27	3.70	16
F_2_	0.36 (0.26–0.45)	0.54 (0.42–0.64)	0.83 (0.70–0.96)	1.92 (1.69–2.23)	3.54 ± 0.17	61.57	3.84	16
Third instar								
F_0_	1.08 (0.85–1.30)	1.55 (1.30–1.76)	2.29 (2.06–2.50)	4.83 (4.41–5.43)	3.95 ± 0.25	40.88	2.55	16
F_1_	0.99 (0.76–1.19)	1.42 (1.18–1.63)	2.13 (1.90–2.33)	4.58 (4.20–5.12)	3.86 ± 0.26	36.34	2.27	16
F_2_	0.65 (0.51–0.78)	1.06 (0.90–1.21)	1.85 (1.66–2.03)	5.23 (4.60–6.13)	2.83 ± 0.12	54.21	3.38	16
Fourth instar								
F_0_	0.88 (0.69–1.06)	1.44 (1.22–1.63)	2.47 (2.23–2.73)	6.95 (5.94–8.53)	2.86 ± 0.13	65.39	4.08	16
F_1_	0.80 (0.62–0.96)	1.33 (1.12–1.51)	2.32 (2.08–2.56)	6.73 (5.76–8.25)	2.77 ± 0.12	63.76	3.98	16
F_2_	0.81 (0.63–0.97)	1.34 (1.14–1.52)	2.35 (2.12–2.59)	6.83 (5.85–8.35)	2.76 ± 0.13	61.22	3.82	16
Fifth instar								
F_0_	1.39 (1.23–1.53)	1.97 (1.81–2.11)	2.89 (2.75–3.03)	6.02 (5.66–6.48)	4.03 ± 0.25	14.38	0.89	16
F_1_	1.49 (1.31–1.65)	2.05 (1.88–2.21)	2.93 (2.77–3.07)	5.74 (5.40–6.18)	4.38 ± 0.30	16.86	1.05	16
F_2_	1.48 (1.27–1.66)	2.05 (1.85–2.23)	2.96 (2.79–3.12)	5.94 (5.53–6.48)	4.24 ± 0.30	20.33	1.27	16
Sixth instar								
F_0_	1.41 (1.07–1.69)	2.34 (2.02–2.60)	4.11 (3.97–4.54)	12.01 (9.49–17.26)	2.75 ± 0.22	38.21	2.38	16
F_1_	1.05 (0.82–1.27)	2.00 (1.73–2.25)	4.08 (3.65–4.64)	15.77 (12.09–22.86)	2.18 ± 0.12	47.73	2.98	16
F_2_	1.31 (0.93–1.62)	2.25 (1.885–2.54)	4.10 (3.73–4.62)	12.82 (9.75–20.07)	2.59 ± 0.21	48.36	3.02	16

The values in parentheses present the range of the respective means; values are means ± SE (standard errors of the means).

**Table 2:** Sub-lethal (LC_10_, LC_25_) effects of chlorantraniliprole on biological traits of *Spodoptera frugiperda* for three generations.

Conc.	Duration (Egg- Larva) (Days)	1st Instar	2nd Instar	3rd Instar	4th Instar	5th Instar	6th Instar	Pupa
F_0_								
Control	2.80 ± 0.04	3.18 ± 0.04	1.26 ± 0.04	1.32 ± 0.04	1.26 ± 0.04	2.19 ± 0.04	3.12 ± 0.03	8.68 ± 0.06
LC_10_	3.28 ± 0.04	3.42 ± 0.05	1.40 ± 0.05	1.51 ± 0.05	1.56 ± 0.05	2.53 ± 0.05	3.69 ± 0.04	9.57 ± 0.07
LC_25_	3.65 ± 0.04	3.93 ± 0.02	1.92 ± 0.02	1.80 ± 0.04	1.82 ± 0.03	2.81 ± 0.04	3.93 ± 0.02	10.17 ± 0.07
*p*-value	0.21	0.000124	0.0152	0.088	0.0037	0.013	0.0009	0.243
*F*	6.40	8.92	4.43	9.75	2.78	1.85	4.42	1.39
df	89	89	89	89	89	89	89	89
F_1_								
Control	2.68 ± 0.05	3.12 ± 0.03	1.23 ± 0.04	1.31 ± 0.04	1.28 ± 0.04	2.27 ± 0.04	3.10 ± 0.03	8.65 ± 0.07
LC_10_	3.45 ± 0.05	3.48 ± 0.05	1.38 ± 0.05	1.52 ± 0.05	1.40 ± 0.05	2.62 ± 0.05	3.70 ± 0.04	9.77 ± 0.08
LC_25_	3.87 ± 0.03	3.95 ± 0.02	1.89 ± 0.03	1.86 ± 0.03	1.93 ± 0.02	2.86 ± 0.03	3.95 ± 0.02	9.94 ± 0.09
*p*-value	0.205	0.066	0.010	0.024	0.384	0.011	0.26	0.646
*F*	5.45	2.79	4.21	9.56	3.83	1.89	1.23	0.51
df	89	89	89	89	89	89	89	89
F_2_								
Control	2.54 ± 0.05	3.07 ± 0.02	1.07 ± 0.02	1.36 ± 0.05	1.25 ± 0.04	2.19 ± 0.04	3.07 ± 0.02	8.79 ± 0.06
LC_10_	3.39 ± 0.05	3.22 ± 0.04	1.45 ± 0.05	1.50 ± 0.50	1.54 ± 0.05	2.62 ± 0.05	3.55 ± 0.05	9.65 ± 0.07
LC_25_	3.76 ± 0.04	3.92 ± 0.02	1.93 ± 0.02	1.88 ± 0.03	1.90 ± 0.03	2.87 ± 0.03	3.93 ± 0.02	9.88 ± 0.08
*p*-value	0.65	0.123	0.989	0.94	0.007	0.068	0.20	0.130
*F*	0.63	0.28	4.25	10.70	2.48	3.14	1.93	0.36
df	89	89	89	89	89	89	89	89

Values are means ± SE (standard errors of the means).

**Table 3:** Sub-lethal (LC_10_, LC_25_) effects of chlorantraniliprole on reproductive parameters of *Spodoptera frugiperda* for 3 generations.

Concentration	Pre-Oviposition Period (Days)	Fecundity	Female Adult Longevity (Days)
F_0_			
Control	3.36 ± 0.065	394.53 ± 5.74	4.519 ± 0.067
LC_10_	3.53 ± 0.068	341.73 ± 6.17	4.538 ± 0.068
LC_25_	3.65 ± 0.065	337.15 ± 6.19	4.576 ± 0.067
*p*-value	0.523	0.991	0.368
*F*	0.955	1.738	0.412
F_1_			
Control	3.30 ± 0.063	368.71 ± 6.16	4.480 ± 0.067
LC_10_	3.42 ± 0.067	343.38 ± 5.013	4.384 ± 0.066
LC_25_	3.59 ± 0.067	346.76 ± 5.92	4.461 ± 0.068
*p*-value	0.0032	0.172	0.468
*F*	3.27	1.390	0.095
F_2_			
Control	3.28 ± 0.062	368.38 ± 5.535	4.384 ± 0.066
LC_10_	3.44 ± 0.068	361.80 ± 5.799	4.423 ± 0.067
LC_25_	3.69 ± 0.063	329.07 ± 5.177	4.403 ± 0.067
*p*-value	0.00040	0.284	0.943
*F*	3.61	0.295	1.894

Values are means ± SE (standard errors of the means).

**Table 4:** Transgenerational effects of chlorantraniliprole on demographic traits of *Spodoptera frugiperda* for the F_0_, F_1_, and F_2_ generations.

F_0_			
	LC_10_	Control	LC_25_
r	0.172 ± 0.0036 ab	0.194 ± 0.0040 a	0.160 ± 0.0034 b
λ	1.188 ± 0.0043 ab	1.215 ± 0.0048 a	1.174 ± 0.0039 b
R_0_	185.544 ± 18.16 b	206.19 ± 21.006 a	196.02 ± 18.074 b
T	30.259 ± 0.26 a	27.347 ± 0.184 b	32.903 ± 0.375 a
GRR	196.990 ± 18.24 b	214.849 ± 21.13 a	211.319 ± 18.43 b
F_1_			
r	0.170 ± 0.0036 ab	0.199 ± 0.0036 a	0.157 ± 0.0029 ab
λ	1.186 ± 0.0042 ab	1.220 ± 0.0044 a	1.170 ± 0.0034 ab
R_0_	182.322 ± 18.26 b	213.33 ± 19.597 a	196.122 ± 18.34 b
T	30.472 ± 0.232 a	26.866 ± 0.173 b	33.564 ± 0.153 a
GRR	198.209 ± 18.702 b	220.959 ± 19.69 a	215.759 ± 18.82 ab
F_2_			
r	0.183 ± 0.0049 b	0.211 ± 0.0062 a	0.158 ± 0.0029 b
λ	1.201 ± 0.0059 a	1.235 ± 0.0077 a	1.171 ± 0.0034 b
R_0_	213.77 ± 19.11 ab	217.34 ± 19.35 a	193.83 ± 17.30 b
T	29.26 ± 0.599 a	25.42 ± 0.601 b	33.266 ± 0.265 a
GRR	226.73 ± 19.48 ab	229.19 ± 19.65 a	201.04 ± 17.47 b

Here, r—intrinsic rate of increase, λ—finite rate of increase, R_0_—net reproduction rate, T—mean length of a generation, GRR—gross reproduction rate; values are means ± SE (standard errors of the means).The means followed by different letters are significantly different from each other (*p* < 0.05)

## Data Availability

All data are within the manuscript.

## References

[1] Song X.-P., Liang Y.-J., Zhang X.-Q., Qin Z.-Q., Wei J.-J., Li Y.-R., Wu J.-M. (2020). Intrusion of Fall Armyworm (*Spodoptera frugiperda*) in Sugarcane and Its Control by Drone in China. Sugar Tech.

[2] Sparks A.N. (1979). A Review of the Biology of the Fall Armyworm. Fla. Entomol..

[3] Goergen G., Kumar P.L., Sankung S.B., Togola A., Tamò M. (2016). First Report of Outbreaks of the Fall Armyworm *Spodoptera frugiperda* (JE Smith) (Lepidoptera, Noctuidae), a New Alien Invasive Pest in West and Central Africa. PLoS ONE.

[4] Silva A.A., Alvarenga R., Moraes J.C., Alcantra E. (2014). Biologia de *Spodoptera frugiperda* (JE Smith) (Lepidoptera: Noctuidae) Em Algodoeiro de Fibra Colorida Tratado Com Silício. EntomoBrasilis.

[5] Ali S., Li Y., Haq I.U., Abbas W., Shabbir M.Z., Khan M.M., Mamay M., Niaz Y., Farooq T., Skalicky M. (2021). The Impact of Different Plant Extracts on Population Suppression of *Helicoverpa armigera* (Hub.) and Tomato (Lycopersicon Esculentum Mill) Yield under Field Conditions. PLoS ONE.

[6] Zaimi S., Saranum M., Hudin L., Ali W. (2021). First Incidence of the Invasive Fall Armyworm, *Spodoptera frugiperda* (J.E. Smith, 1797) Attacking Maize in Malaysia. BioInvasions Rec..

[7] Boregas K.G.B., Mendes S.M., Waquil J.M., Fernandes G.W. (2013). Estádio de Adaptação de *Spodoptera frugiperda* (JE Smith) (Lepidoptera: Noctuidae) Em Hospedeiros Alternativos. Bragantia.

[8] Sisay B., Tefera T., Wakgari M., Ayalew G., Mendesil E. (2019). The Efficacy of Selected Synthetic Insecticides and Botanicals against Fall Armyworm, *Spodoptera frugiperda*, in Maize. Insects.

[9] Idrees A., Qadir Z.A., Afzal A., Ranran Q., Li J. (2022). Laboratory Efficacy of Selected Synthetic Insecticides against Second Instar Invasive Fall Armyworm, *Spodoptera frugiperda* (Lepidoptera: Noctuidae) Larvae. PLoS ONE.

[10] Casida J.E., Durkin K.A. (2013). Neuroactive Insecticides: Targets, Selectivity, Resistance, and Secondary Effects. Annu Rev Entomol.

[11] Casida J.E. (2009). Pest Toxicology: The Primary Mechanisms of Pesticide Action. Chem. Res. Toxicol..

[12] Wu H.-M., Feng H.-L., Wang G.-D., Zhang L.-L., Zulu L., Liu Y.-H., Zheng Y.-L., Rao Q. (2022). Sublethal Effects of Three Insecticides on Development and Reproduction of *Spodoptera frugiperda* (Lepidoptera: Noctuidae). Agronomy.

[13] Tulashie S.K., Adjei F., Abraham J., Addo E. (2021). Potential of Neem Extracts as Natural Insecticide against Fall Armyworm (*Spodoptera frugiperda* (J. E. Smith) (Lepidoptera: Noctuidae). Case Stud. Chem. Environ. Eng..

[14] Kulye M., Mehlhorn S., Boaventura D., Godley N., Venkatesh S., Rudrappa T., Charan T., Rathi D., Nauen R. (2021). Baseline Susceptibility of *Spodoptera frugiperda* Populations Collected in India towards Different Chemical Classes of Insecticides. Insects.

[15] Ahissou B.R., Sawadogo W.M., Bokonon-Ganta A.H., Somda I., Kestemont M.-P., Verheggen F.J. (2021). Baseline Toxicity Data of Different Insecticides against the Fall Armyworm *Spodoptera frugiperda* (J.E. Smith) (Lepidoptera: Noctuidae) and Control Failure Likelihood Estimation in Burkina Faso. Afr. Entomol..

[16] Rajula J., Pittarate S., Suwannarach N., Kumla J., Ptaszynska A.A., Thungrabeab M., Mekchay S., Krutmuang P. (2021). Evaluation of Native Entomopathogenic Fungi for the Control of Fall Armyworm (*Spodoptera frugiperda*) in Thailand: A Sustainable Way for Eco-Friendly Agriculture. J. Fungi.

[17] Osae M.Y., Frimpong J.O., Sintim J.O., Offei B.K., Marri D., Ofori S.E.K. (2022). Evaluation of Different Rates of Ampligo Insecticide against Fall Armyworm (*Spodoptera frugiperda* (J.E. Smith); Lepidoptera: Noctuidae) in the Coastal Savannah Agroecological Zone of Ghana. Adv. Agric..

[18] Deshmukh S., Pavithra H.B., Kalleshwaraswamy C.M., Shivanna B.K., Maruthi M.S., Mota-Sanchez D. (2020). Field Efficacy of Insecticides for Management of Invasive Fall Armyworm, *Spodoptera frugiperda* (J.E. Smith) (Lepidoptera: Noctuidae) on Maize in India. Fla. Entomol..

[19] Hardke J.T., Temple J.H., Leonard B.R., Jackson R.E. (2011). Laboratory Toxicity and Field Efficacy of Selected Insecticides Against Fall Armyworm (Lepidoptera: Noctuidae) 1. Fla. Entomol..

[20] Adamczyk J.J., Leonard B.R., Graves J.B. (1999). Toxicity of Selected Insecticides to Fall Armyworms (Lepidoptera: Noctuidae) in Laboratory Bioassay Studies. Fla. Entomol..

[21] Lai T., Su J. (2011). Effects of Chlorantraniliprole on Development and Reproduction of Beet Armyworm, Spodoptera Exigua (Hübner). J. Pest Sci..

[22] Cao G., Lu Q., Zhang L., Guo F., Liang G., Wu K., Wyckhuys K.A.G., Guo Y. (2010). Toxicity of Chlorantraniliprole to Cry1Ac-Susceptible and Resistant Strains of Helicoverpa Armigera. Pestic. Biochem. Physiol..

[23] Liu Z.-K., Li X.-L., Tan X.-F., Yang M.-F., Idrees A., Liu J.-F., Song S.-J., Shen J. (2022). Sublethal Effects of Emamectin Benzoate on Fall Armyworm, *Spodoptera frugiperda* (Lepidoptera: Noctuidae). Agriculture.

[24] Singh J.P., Marwaha K.K. (2000). Effect of Sublethal Concentrations of Some Insecticides on Growth and Development of Maize Stalk Borer, Chilo Partellus (Swinhoe) Larvae. Shashpa.

[25] Smagghe G., Tirry L. (2001). Insect Midgut as a Site for Insecticide Detoxification and Resistance. Biochemical Sites of Insecticide Action and Resistance.

[26] Cutler G.C. (2013). Insects, Insecticides and Hormesis: Evidence and Considerations for Study. Dose-Response.

[27] Haynes K.F. (1988). Sublethal Effects of Neurotoxic Insecticides on Insect Behavior. Annu. Rev. Entomol..

[28] Toscano L.C., Fernandes M.A., Rota M., Maruyama W.I., Andrade J.V. (2016). híBridos de Milho Frente Ao Ataque De *Spodoptera frugiperda* em Associação Com Adubação Silicatada E O Efeito Sobre O Predador Doru Luteipes. Rev. Agric. Neotrop..

[29] Teke M.A., Mutlu Ç. (2021). Insecticidal and Behavioral Effects of Some Plant Essential Oils against Sitophilus Granarius L. and Tribolium Castaneum (Herbst). J. Plant Dis. Prot..

[30] Wang Y., Ma Q., Tan Y., Zheng Q., Yan W., Yang S., Xu H., Zhang Z. (2019). The Toxicity and Field Efficacy of Chlorantraniliprole against *Spodoptera frugiperda*. J. Environ. Entomol..

[31] Li X., Jiang H., Wu J., Zheng F., Xu K., Lin Y., Zhang Z., Xu H. (2021). Drip Application of Chlorantraniliprole Effectively Controls Invasive *Spodoptera frugiperda* (Lepidoptera: Noctuidae) and Its Distribution in Maize in China. Crop Prot..

[32] Pes M.P., Melo A.A., Stacke R.S., Zanella R., Perini C.R., Silva F.M.A., Carús Guedes J.V. (2020). Translocation of Chlorantraniliprole and Cyantraniliprole Applied to Corn as Seed Treatment and Foliar Spraying to Control *Spodoptera frugiperda* (Lepidoptera: Noctuidae). PLoS ONE.

[33] Wang Q., Rui C., Wang L., Huang W., Zhu J., Ji X., Yang Q., Liang P., Yuan H., Cui L. (2022). Comparative Toxicity and Joint Effects of Chlorantraniliprole and Carbaryl Against the Invasive Spodioptera Frugiperda (Lepidoptera: Noctuidae). J. Econ. Entomol..

[34] Altaf N., Arshad M., Majeed M.Z., Ullah M.I., Latif H., Zeeshan M., Yousuf G., Afzal M. (2022). Comparative Effectiveness of Chlorantraniliprole and Neem Leaf Extract against Fall Armyworm, *Spodoptera frugiperda* (JE Smith) (Lepidoptera: Noctuidae). Sarhad J. Agric..

[35] Ahn J.J., Choi K.S. (2022). Population Parameters and Growth of Riptortus Pedestris (Fabricius) (Hemiptera: Alydidae) under Fluctuating temperature. Insects.

[36] Berber G., Birgücü A.K. (2022). Effects of Two Different Isolates of Entomopathogen Fungus, Beauveria Bassiana (Balsamo) Vuillemin on Myzus Persicae Sulzer (Hemiptera: Aphididae). Tarım Bilim. Derg..

[37] Idrees A., Qadir Z.A., Akutse K.S., Afzal A., Hussain M., Islam W., Waqas M.S., Bamisile B.S., Li J. (2021). Effectiveness of Entomopathogenic Fungi on Immature Stages and Feeding Performance of Fall Armyworm, *Spodoptera frugiperda* (Lepidoptera: Noctuidae) Larvae. Insects.

[38] Idrees A., Afzal A., Qadir Z.A., Li J. (2022). Bioassays of Beauveria Bassiana Isolates against the Fall Armyworm, *Spodoptera frugiperda*. J. Fungi.

[39] Younas H., Razaq M., Farooq M.O., Saeed R. (2022). Host Plants of Phenacoccus Solenopsis (Tinsley) Affect Parasitism of Aenasius Bambawalei (Hayat). Phytoparasitica.

[40] Abdel-Khalek A.A., Momen F.M. (2022). Biology and Life Table Parameters of Proprioseiopsis Lindquisti on Three Eriophyid Mites (Acari: Phytoseiidae: Eriophyidae). Persian J. Acarol..

[41] Huang Y.-B., Chi H. (2011). The Age-Stage, Two-Sex Life Table with an Offspring Sex Ratio Dependent on Female Age. J. Agric..

[42] Chi H. (1988). Life-Table Analysis Incorporating Both Sexes and Variable Development Rates among Individuals. Environ. Entomol..

[43] Zheng X.-M., Tao Y.-L., Chi H., Wan F.-H., Chu D. (2017). Adaptability of Small Brown Planthopper to Four Rice Cultivars Using Life Table and Population Projection Method. Sci. Rep..

[44] Planes L., Catalán J., Tena A., Porcuna J.L., Jacas J.A., Izquierdo J., Urbaneja A. (2013). Lethal and Sublethal Effects of Spirotetramat on the Mealybug Destroyer, Cryptolaemus Montrouzieri. J. Pest Sci..

[45] Sunarto D.A. (2009). Peran Insektisida Botani Ekstrak Biji Mimba Untuk Konservasi Musuh Alami Dalam Pengelolaan Serangga Hama Kapas. J. Entomol. Indones..

[46] Xie W., Zhi J., Ye J., Zhou Y., Li C., Liang Y., Yue W., Li D., Zeng G., Hu C. (2021). Age-Stage, Two-Sex Life Table Analysis of *Spodoptera frugiperda* (JE Smith) (Lepidoptera: Noctuidae) Reared on Maize and Kidney Bean. Chem. Biol. Technol. Agric..

[47] Guo J., Zhang M., Gao Z., Wang D., He K., Wang Z. (2021). Comparison of Larval Performance and Oviposition Preference of *Spodoptera frugiperda* among Three Host Plants: Potential Risks to Potato and Tobacco Crops. Insect Sci..

[48] Wu L., Zhou C., Long G., Yang X., Wei Z., Liao Y., Yang H., Hu C. (2021). Fitness of Fall Armyworm, *Spodoptera frugiperda* to Three Solanaceous Vegetables. J. Integr. Agric..

[49] He L., Wu Q., Gao X., Wu K. (2021). Population Life Tables for the Invasive Fall Armyworm, *Spodoptera frugiperda* Fed on Major Oil Crops Planted in China. J. Integr. Agric..

[50] Gao Z., Chen Y., He K., Guo J., Wang Z. (2021). Sublethal Effects of the Microbial-Derived Insecticide Spinetoram on the Growth and Fecundity of the Fall Armyworm (Lepidoptera: Noctuidae). J. Econ. Entomol..

[51] Iqbal H., Fatima A., Khan H.A.A. (2022). ZnO nanoparticles produced in the culture supernatant of *Bacillus thuringiensis* ser. israelensis affect the demographic parameters of *Musca domestica* using the age-stage, two-sex life table. Pest Manag. Sci..

[52] Rehman S.U., Zhou X., Ali S., Rasheed M.A., Islam Y., Hafeez M., Sohail M.A., Khurram H. (2020). Predatory Functional Response and Fitness Parameters of Orius Strigicollis Poppius When Fed Bemisia Tabaci and Trialeurodes Vaporariorum as Determined by Age-Stage, Two-Sex Life Table. PeerJ.

[53] Alinejad M., Kheradmand K., Fathipour Y. (2020). Demographic Analysis of Sublethal Effects of Propargite on Amblyseius Swirskii (Acari: Phytoseiidae): Advantages of Using Age-Stage, Two Sex Life Table in Ecotoxicological Studies. Syst. Appl. Acarol..

[54] Shahzad M.F., Idrees A., Afzal A., Iqbal J., Qadir Z.A., Khan A.A., Ullah A., Li J. (2022). RNAi-Mediated Silencing of Putative Halloween Gene Phantom Affects the Performance of Rice Striped Stem Borer, Chilo Suppressalis. Insects.

[55] Ahmed K.S., Idrees A., Majeed M.Z., Majeed M.I., Shehzad M.Z., Ullah M.I., Afzal A., Li J. (2022). Synergized Toxicity of Promising Plant Extracts and Synthetic Chemicals against Fall Armyworm *Spodoptera frugiperda* (JE Smith) (Lepidoptera: Noctuidae) in Pakistan. Agronomy.

[56] Qadir Z.A., Idrees A., Mahmood R., Sarwar G., Bakar M.A., Ahmad S., Raza M.M., Li J. (2021). Effectiveness of Different Soft Acaricides against Honey Bee Ectoparasitic Mite *Varroa destructor* (Acari: Varroidae). Insects.

[57] Idrees A. (2017). Protein Baits, Volatile Compounds And Irradiation Influence The Expression Profiles Of Odorantbinding Protein Genes in *Bactrocera dorsalis* (Diptera: Tephritidae). Appl. Ecol. Environ. Res..

[58] Idrees A., Qasim M., Ali H., Qadir Z.A., Idrees A., Bashir M.H., Qing J.E. (2016). Acaricidal Potential of Some Botanicals against the Stored Grain Mites, *Rhizoglyphus tritici*. J. Entomol. Zool. Stud..

[59] Robertson J.L., Jones M.M., Olguin E., Alberts B. (2017). Bioassays with Arthropods.

[60] SAS Institute Inc. SAS Software 9.4. TWOSEX-MSChart. SAS Inst. Inc.: Cary, NC, USA, 2014.

[61] Chi H. TWOSEX-MSChart: A Computer Program for the Age-Stage, Two-Sex Life Table Analysis. http://140.120.197.173/Ecology/prod02.htm.

[62] Huang Y., Chi H. (2012). Age-stage, Two-sex Life Tables of Bactrocera Cucurbitae (Coquillett) (Diptera: Tephritidae) with a Discussion on the Problem of Applying Female Age-specific Life Tables to Insect Populations. Insect Sci..

[63] Akkopru E.P., Atl han R., Okut H., Chi H. (2015). Demographic Assessment of Plant Cultivar Resistance to Insect Pests: A Case Study of the Dusky-Veined Walnut Aphid (Hemiptera: Callaphididae) on Five Walnut Cultivars. J. Econ. Entomol..

[64] Goodman D. (1982). Optimal Life Histories, Optimal Notation, and the Value of Reproductive Value. Am. Nat..

[65] Chi H., Getz W.M. (1988). Mass Rearing and Harvesting Based on an Age-Stage, Two-Sex Life Table: A Potato Tuberworm (Lepidoptera: Gelechiidae) Case Study. Environ. Entomol..

[66] Yang Y., Li W., Xie W., Wu Q., Xu B., Wang S., Li C., Zhang Y. (2015). Development of Bradysia Odoriphaga (Diptera: Sciaridae) as Affected by Humidity: An Age–Stage, Two-Sex, Life-Table Study. Appl. Entomol. Zool..

[67] Harcourt D.G. (1969). The Development and Use of Life Tables in the Study of Natural Insect Populations. Annu. Rev. Entomol..

[68] Rozilawati H., Mohd Masri S., Tanaselvi K., Mohd Zahari T.H., Zairi J., Nazni W.A., Lee H.L. (2018). Life Table Characteristics of Malaysian Strain Aedes Albopictus (Skuse). Serangga.

[69] Teixeira L.A.F., Gut L.J., Wise J.C., Isaacs R. (2009). Lethal and Sublethal Effects of Chlorantraniliprole on Three Species of Rhagoletis Fruit Flies (Diptera: Tephritidae). Pest Manag. Sci..

[70] Knight A.L., Flexner L. (2007). Disruption of Mating in Codling Moth (Lepidoptera: Tortricidae) by Chlorantranilipole, an Anthranilic Diamide Insecticide. Pest Manag. Sci..

[71] Han W., Zhang S., Shen F., Liu M., Ren C., Gao X. (2012). Residual Toxicity and Sublethal Effects of Chlorantraniliprole on Plutella Xylostella (Lepidoptera: Plutellidae). Pest Manag. Sci..

[72] Lutz A.L., Bertolaccini I., Scotta R.R., Curis M.C., Favaro M.A., Fernandez L.N., Sánchez D.E. (2018). Lethal and Sublethal Effects of Chlorantraniliprole on Spodoptera Cosmioides (Lepidoptera: Noctuidae). Pest Manag. Sci..

[73] Nawaz M., Cai W., Jing Z., Zhou X., Mabubu J.I., Hua H. (2017). Toxicity and Sublethal Effects of Chlorantraniliprole on the Development and Fecundity of a Non-Specific Predator, the Multicolored Asian Lady Beetle, Harmonia Axyridis (Pallas). Chemosphere.

[74] Wang X., Li X., Shen A., Wu Y. (2010). Baseline Susceptibility of the Diamondback Moth (Lepidoptera: Plutellidae) to Chlorantraniliprole in China. J. Econ. Entomol..

[75] Sial A.A., Brunner J.F. (2010). Toxicity and Residual Efficacy of Chlorantraniliprole, Spinetoram, and Emamectin Benzoate to Obliquebanded Leafroller (Lepidoptera: Tortricidae). J. Econ. Entomol..

[76] Fernandes F.O., de Souza T.D., Sanches A.C., Dias N.P., Desiderio J.A., Polanczyk R.A. (2021). Sub-Lethal Effects of a Bt-Based Bioinsecticide on the Biological Conditioning of Anticarsia Gemmatalis. Ecotoxicology.

[77] Yin X.H., Wu Q.J., Li X.F., Zhang Y.J., Xu B.Y. (2008). Sublethal Effects of Spinosad on Plutella Xylostella (Lepidoptera: Yponomeutidae). Crop Prot..

